# Familial Spontaneous Keloids: Examining Thoracic Manifestations in Two Brothers

**DOI:** 10.7759/cureus.64163

**Published:** 2024-07-09

**Authors:** Ioannis Kyriazidis, Efterpi Demiri, Pericles Foroglou

**Affiliations:** 1 Department of Plastic and Reconstructive Surgery, General Hospital Papageorgiou, Thessaloniki, GRC; 2 Department of Plastic and Reconstructive Surgery, School of Medicine, Faculty of Health Sciences, Aristotle University of Thessaloniki, Thessaloniki, GRC

**Keywords:** keloid pathogenesis, keloid formation, siblings, thoracic region, genetic predisposition, familial spontaneous keloids, spontaneous keloid, familial keloid, keloids

## Abstract

Keloids are complex fibroproliferative disorders with diverse clinical presentations. Spontaneous keloids (SKs) represent a rare subtype that emerges without any known preceding traumatic event. This report presents a case of familial spontaneous keloids appearing on the thoracic region in two brothers with no prior history of trauma or keloid occurrence in other family members. The lesions exhibited progressive growth over several years but responded to cycles of triamcinolone treatment. This case underscores an unusual spontaneous occurrence of keloids in the thoracic region of two siblings, highlighting the potential genetic predisposition in the aetiology of these lesions. Additionally, this instance reinforces the concept that keloids can develop spontaneously without any apparent trauma in the affected area.

## Introduction

Keloids are characterized by an overactive form of scar tissue development, presenting as thick scars that extend beyond the initial boundaries of the wound. They result from an abnormal healing response, involving excessive growth of fibrous tissue that surpasses the original wound margins [1]. These lesions pose a significant clinical challenge due to their unpredictable growth, high recurrence rates, and resistance to treatment [2]. The underlying pathogenesis of keloids is believed to involve an aberrant wound healing process, marked by the excessive deposition of extracellular matrix components, predominantly collagen [1].

Spontaneous keloids, a rare subtype, occur without any known preceding trauma, adding complexity to the understanding of keloid formation [3]. The rarity and significant impact of spontaneous and familial keloids necessitate further investigation. Previous literature reviews have highlighted the unusual occurrence and potential genetic predisposition associated with these lesions [4]. This report presents cases of familial spontaneous keloids in the thoracic region of two brothers, emphasizing the potential genetic factors involved in their development and contributing to the broader understanding of keloid pathogenesis.

## Case presentation

**Figure 1:**
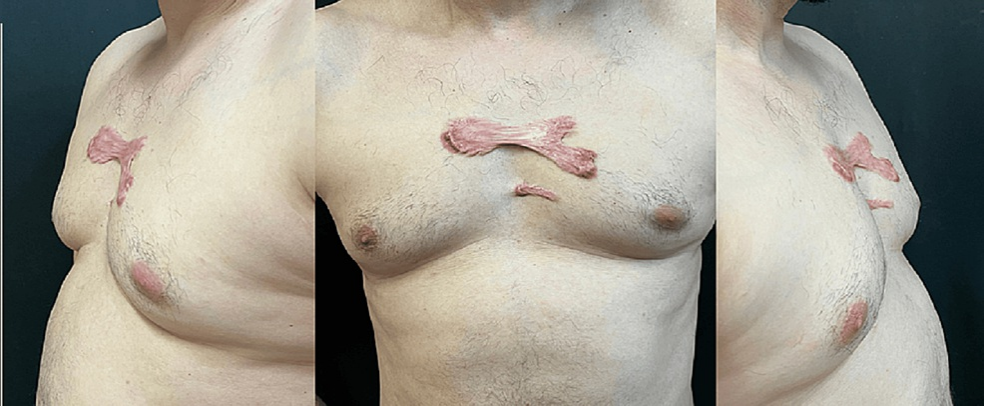
Clinical presentation of spontaneous keloids on the sternal region of a 41-year-old male patient The images depict the keloid lesions from multiple angles, illustrating their size, shape, and distribution. The keloids developed without any preceding trauma or identifiable aggravating factors, suggesting a potential genetic predisposition.

A 41-year-old Caucasian (Albanian) male manual worker with Fitzpatrick skin type 3 presented with keloid formations on his sternal area, devoid of any antecedent skin trauma or identifiable aggravating factors. The initial keloid appeared in 2009, followed by the emergence of a second lesion in 2012. These lesions subsequently converged to form an enlarged keloid (Figure 1).

**Figure 2:**
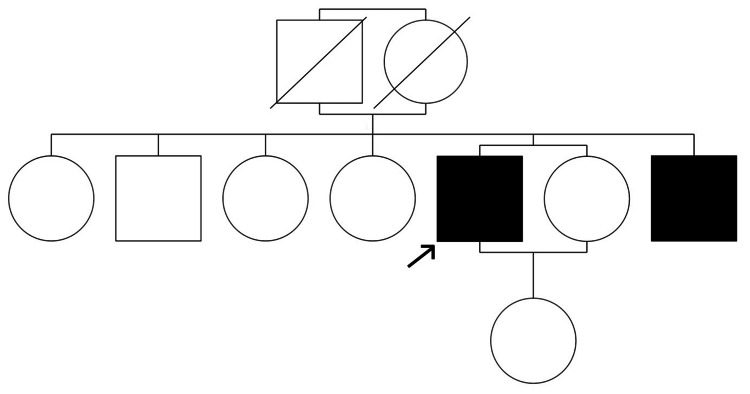
Pedigree chart depicting familial keloid manifestation in our patient's family

Despite the patient's history of diverse skin injuries, including lacerations on his forearms from a sharp instrument and a brick, none of these injuries culminated in keloid formation. This inconsistency between injury and keloidogenesis underscores the complexity of keloid pathogenesis and potentially challenges the mechanical tension hypothesis in this context. The patient’s family history is notable; he originates from a large family (Figure 2), including three sisters and two brothers.

**Figure 3:**
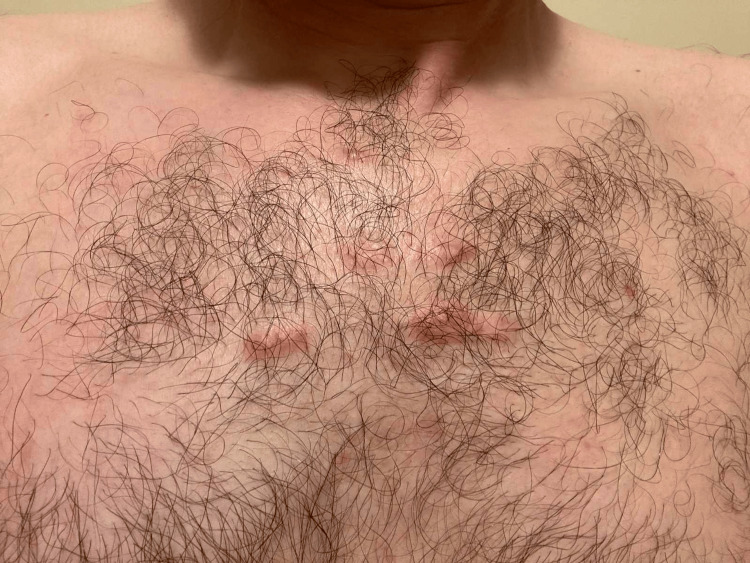
Multiple keloid scars on the chest of the patient's youngest brother The lesions developed spontaneously without any preceding trauma, further supporting the hypothesis of a genetic predisposition to keloid formation within this family.

Among them, only his youngest brother exhibits a similar pattern of spontaneous, trauma-independent keloid formation on his chest (Figure 3). Interestingly, the remaining siblings, despite reporting traumatic incidents, have not developed keloids. Additionally, his child has not developed keloids to date. This familial pattern, characterized by a dichotomous response to skin injury and trauma-independent keloid formation in two siblings, suggests a potential genetic predisposition.

Interestingly, the remaining siblings, despite reporting traumatic incidents, have not developed keloids. Additionally, his child has not developed keloids to date. This familial pattern, characterized by a dichotomous response to skin injury and trauma-independent keloid formation in two siblings, suggests a potential genetic predisposition.

A comprehensive dermatological examination of both the patient and his brother revealed no signs of common skin conditions often linked to keloid development, such as acne keloidalis nuchae, isolated or spontaneous folliculitis, or folliculitis due to chest shaving or acne. The absence of acneiform eruptions, follicular papules, pustules, or hair follicle inflammation, supported by the patient's medical history and lack of reported symptoms indicative of these conditions, reinforces the presumption that the observed keloids are indeed spontaneous.

Histological examination of the lesion confirmed characteristic features of keloid, including hypercellularity, abnormal collagen deposition, increased vascularization, and the presence of inflammatory cells. These findings reinforce the clinical diagnosis and enhance understanding of the complex pathophysiological processes involved in the spontaneous formation of keloids in these patients.

The lesions responded to cycles of intralesional triamcinolone treatment. The treatment protocol consisted of intralesional injections of triamcinolone acetonide (TCA). We used a 40 mg/mL concentration of TCA (Kenalog-40®), administered at a dosage of 10 mg per square centimeter of keloid tissue. The injections were performed using a 25-gauge needle, ensuring even distribution throughout the lesion. Treatments were administered at four-week intervals for a total duration of six months. Each session involved multiple injections to cover the entire keloid area adequately. The total volume injected per session varied based on the size of the keloid, ranging from 0.5 to 2 mL. We observed the patients for immediate complications such as pain or blanching after each injection. Long-term side effects, including skin atrophy and telangiectasia, were monitored during follow-up visits, but never actually observed. Adjunct treatments, including silicone sheets, were recommended for use between injection sessions to enhance the overall efficacy of the treatment; however, the patient declined these additional interventions. After six cycles of TCA injections, the keloid significantly flattened.

## Discussion

The etiology of keloids involves a complex interplay of genetic, environmental, and systemic factors [2]. Recent advances in keloid research have shed new light on the complex pathophysiology and genetic factors involved in keloid formation, which may help explain the familial occurrence observed in our case. These advances have transformed our perspective from purely fibroproliferative models to more complex, multifactorial paradigms. The tension-induced keloid formation theory proposed by Ogawa et al. suggests that mechanical forces play a crucial role in keloid development, which may be particularly relevant in the thoracic region where skin tension is high [5]. This theory could explain the localization of keloids in our patients, given the mechanical stress experienced in the sternal area.

Additionally, the inflammatory hypothesis posits that chronic inflammation in the reticular dermis drives keloid formation [2]. This is supported by the upregulation of pro-inflammatory cytokines such as interleukin (IL)-1α, IL-1β, IL-6, and tumour necrosis factor (TNF)-α in keloid tissues [6]. The interplay between mechanical stress and inflammation may create a self-perpetuating cycle that promotes keloid growth, particularly in genetically susceptible individuals.

On the genetic front, genome-wide association studies have identified several susceptibility loci, including NEDD4, FOXL2, and PAI [7,8]. These genes are involved in various cellular processes, including extracellular matrix production, cell proliferation, and apoptosis, all of which are dysregulated in keloid formation. The familial occurrence in our case strongly suggests a genetic component, aligning with these findings.

Epigenetic factors, such as altered DNA methylation patterns and histone modifications, have also been implicated in keloid pathogenesis [9]. These epigenetic changes can affect gene expression without altering the DNA sequence, potentially explaining the variable expressivity often observed in familial keloid cases. This could account for the differences in keloid severity and timing of onset between the two brothers in our study.

Recent research has also highlighted the role of the mechanotransduction pathway in keloid formation. Activation of mechanosensitive ion channels and focal adhesion kinases can trigger intracellular signaling cascades that promote fibrosis [10]. This mechanism might be particularly relevant in explaining the spontaneous nature of the keloids in our patients, as even subtle mechanical stresses could potentially trigger keloid formation in genetically predisposed individuals.

Moreover, the concept of keloid stem cells has gained traction, with studies suggesting that a subpopulation of cells with stem cell-like properties may contribute to the continuous growth and recurrence of keloids [11]. This theory could explain the progressive growth of keloids observed in our patients over several years. In the context of our familial case, these genetic, epigenetic, and cellular factors may explain the predisposition to keloid formation in these siblings, despite the absence of apparent trauma. The asynchronous development of keloids in the brothers could be attributed to variable expressivity of the genetic predisposition or differences in environmental triggers.

The genetic analysis of the affected brothers could provide valuable insights into the genetic predisposition associated with spontaneous keloids in this family. Genetic influences on keloid development, although not fully understood, are supported by sporadic cases of familial keloids reported in the literature [12].

Our observations align with other reports of spontaneous keloids in medically healthy individuals. Jfri and Alajmi documented cases of spontaneous keloids on the chest and back in a healthy 21-year-old female and a 39-year-old male, mirroring our patients’ presentation [3]. Similar occurrences have been noted in patients of Syrian and Iraqi origin, reinforcing the sporadic yet significant nature of spontaneous keloids [13,14].

The familial occurrence of keloids in our study is consistent with findings by Marneros and Norris, who suggested an autosomal dominant inheritance pattern with incomplete penetrance and variable expression [15]. Histopathological analysis differentiates keloids from hypertrophic scars by the presence of “keloidal collagen,” characterized by thick eosinophilic collagen bundles [16,17]. Inflammatory factors, such as IL-1α, IL-1β, IL-6, and TNF-α, are upregulated in keloid tissues, suggesting that keloids may be considered inflammatory skin disorders, specifically of the reticular dermis, rather than tumors [6,18].

The spontaneous keloid occurrence in this familial case, independent of known triggering events, indicates a strong genetic predisposition. Previous studies have identified significant correlations between specific Human Leukocyte Antigen (HLA) alleles, such as HLA-DRB1*15 and HLA-DQA1 and DQB1, and keloid formation, suggesting a genetic inclination towards keloid development [7,19]. Clinical data also indicate that individuals with darker skin are more susceptible to pathological scars, including keloids [20].

More recent genetic research has identified several genes, such as SMAD3, EN2, and NDFIP1, that may play crucial roles in keloid pathogenesis [21]. Single nucleotide polymorphisms (SNPs) linked to keloid formation and severity further highlight the genetic complexity of keloid development [22]. Epigenetic factors, including DNA methylation and histone modification, have also been implicated in abnormal wound healing and fibrotic disorders like keloids [23,24].

Molecular mechanisms underlying keloid formation may involve disturbances in growth factor regulation, particularly TGF-B1, which promotes fibroblast proliferation and extracellular matrix synthesis during wound healing [12,25]. The emergence of keloids in the thoracic region in our patients, an area of high skin tension, also indicates the role of mechanical stress as an aetiologic factor. Skin tension might instigate a cascade of cellular and molecular events leading to excessive scar formation [1,2]. Remarkably, studies conducted by Bayat et al. [24] reported a unique pattern in keloid morphology that appeared to be influenced by specific body sites in an African-Caribbean population, underscoring a potential link between keloid manifestation and the location on the body [26]. The emergence of keloids in high-tension areas, such as the thoracic region, suggests that mechanical stress may also play a role in their pathogenesis [1,2]. This insight might provide additional understanding into why our patients developed keloids specifically in the thoracic region, further suggesting that mechanical stress and site-specific factors may play integral roles in keloid pathogenesis.

Several systemic risk factors, including hormonal fluctuations during adolescence and pregnancy, have been associated with increased keloid risk [27,28]. Conditions such as hypertension may exacerbate keloid severity by causing vascular damage and increased local inflammation [29]. Additionally, the role of sex hormones such as oestrogens and androgens in vasodilation and intensifying inflammation may promote pathological scar development or worsen existing scars [30-32]. Systemic inflammation, as observed in disorders like Castleman disease, could also aggravate keloids, providing another layer of complexity to the aetiology of keloids [2].

Our patient’s lesions responded to intralesional triamcinolone, which decreases keloid size by reducing collagen synthesis and increasing collagenase activity [33]. Effective management of keloids remains challenging, with multiple treatment options including corticosteroids, surgery, laser therapy, and radiotherapy [5,33-35]. A tailored approach considering individual patient needs, keloid size, location, and symptoms is essential for effective treatment [29]. For instance, 585-nm flashlamp-pumped pulsed-dye laser treatments have shown promising results in the treatment of hypertrophic and keloidal scars [36]. 

The impact of keloids on quality of life is particularly significant in cases like ours, where the lesions are located in a visible area such as the thoracic region. Both brothers reported feelings of self-consciousness and discomfort when the keloids were visible, affecting their choice of clothing and participation in activities that might expose their chest. This aligns with the findings by Brown et al. [37], who reported that keloids can significantly impact body image and self-esteem.

The familial nature of the condition introduced an additional psychological dimension, with both siblings expressing concern about potential keloid development in other family members, particularly their children. This shared experience, while potentially providing mutual support, also heightened anxiety about the genetic aspects of their condition.

Recent studies have quantified the psychosocial burden of keloids using validated quality-of-life instruments [38]. This study found that patients with keloids scored significantly lower on the Dermatology Life Quality Index compared to the general population, with visible keloids having the most pronounced impact. In our cases, while we did not employ formal quality-of-life assessments, the patient reported improvement in his psychosocial well-being following successful treatment, underscoring the importance of effective keloid management in enhancing overall patient outcomes.

## Conclusions

This case report of familial spontaneous keloids underscores the complexity of keloid pathogenesis and the potential role of genetic predisposition. The development of spontaneous keloids in two brothers, with no apparent external triggers, suggests a strong genetic influence. The characterization of these keloids, along with their histological features, supports the diagnosis and provides insights into the underlying pathophysiological processes.

The labelling of keloids as “spontaneous” does not imply an absence of aetiology but rather an aetiology that is not yet fully understood. Both spontaneous and familial keloids are substantiated by numerous documented cases, highlighting the intricate interplay of genetic, environmental, and systemic factors in their formation. Future research should focus on unravelling the genetic and molecular mechanisms involved in keloid development to improve therapeutic strategies and alleviate the burden of this complex disorder.
